# Supplementary material for: nNOS Increases Fiber Type-Specific Angiogenesis in Skeletal Muscle of Mice in Response to Endurance Exercise
Source: Int J Mol Sci. 2023 May 26;24(11):9341. doi: 10.3390/ijms24119341 (PMC10253500; doi:10.3390/ijms24119341)
Supplement: Supplementary file 1 [file ijms-24-09341-s001.zip › ijms-2390282-supplementary.pdf]

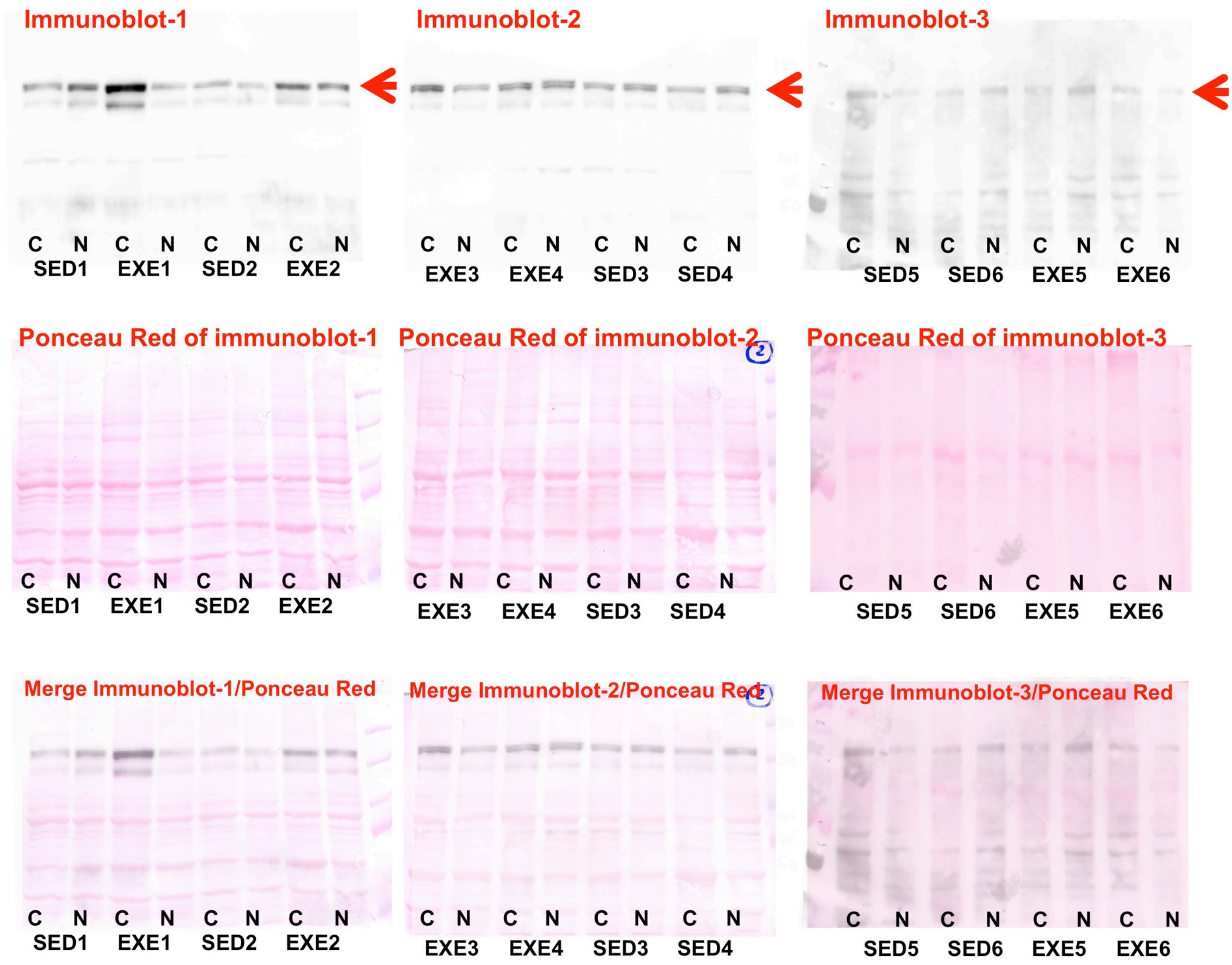

Supplementary Figure S1: nNOS-immunoblotting in solubilsates of transfected TA muscles from trained/sedentary mice.

The TA muscles were subjected to gene electroporation with either the control plasmid without specific insert (C) or the nNOS-plasmid (N) from mice remaining sedentary (SED) or undergoing treadmill exercise (EXE) for seven days. Upper row: On three nitrocellulose matrices, the nNOS-immunoreactive bands are demonstrated as 160 kDa band (red arrows) in the TA muscle solubilsates of the mice from the four study groups (n= 6 mice each). Middle row: After immunoblotting, the blot matrices were subjected to Ponceau Red S-staining for evaluation of protein loading of each lane. Lower row: Overlay/merge of the nNOS-band demonstrated by immunoblotting with the Ponceau Red S-stained nitrocellulose.

Baum et al.

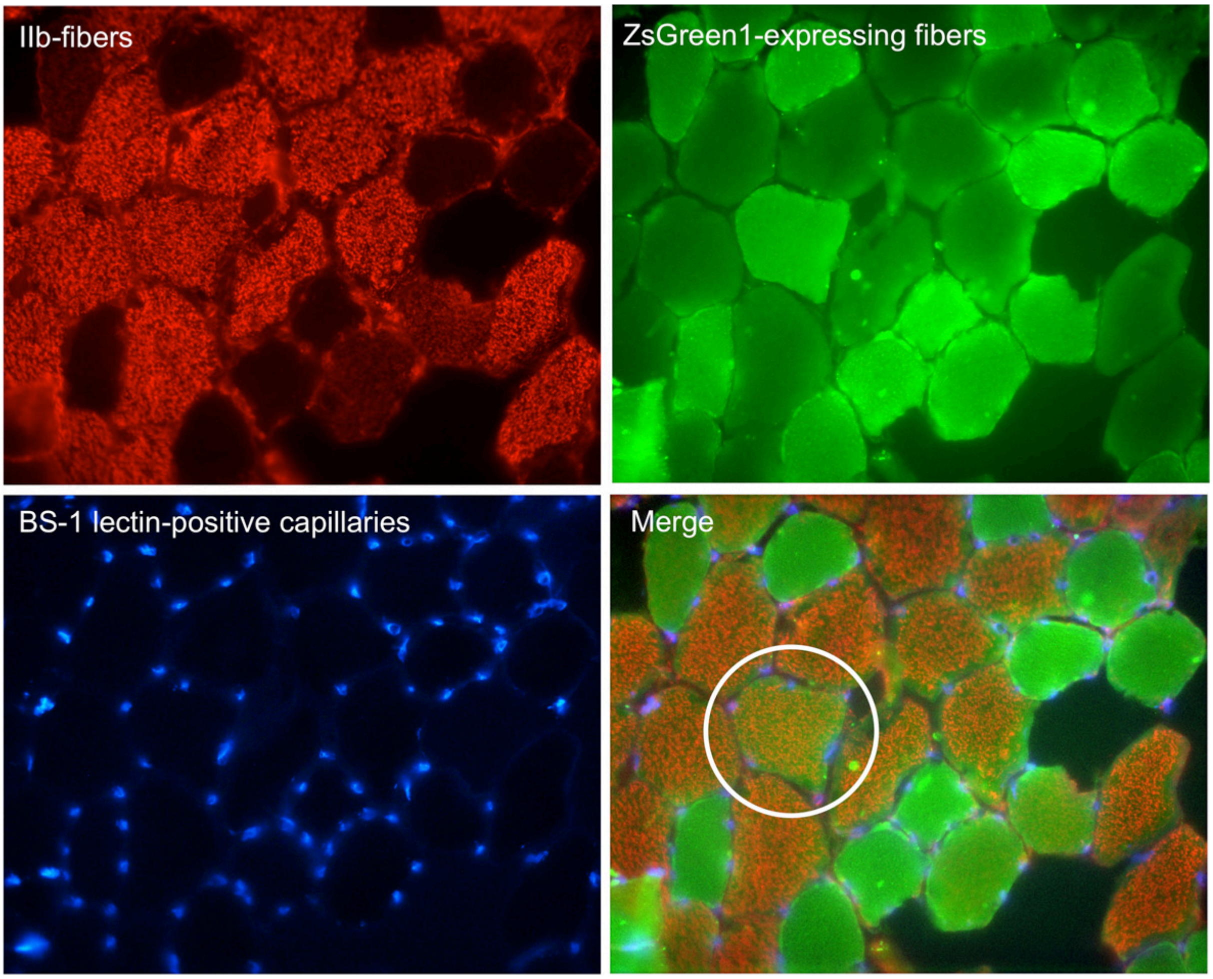

Supplementary Figure S2: An example for the demonstration of three fluorescence photo images used to merge for determination of the index 'capillary contacts' (CC) around skeletal muscle fibers.

Cryosections of the TA from the mice of the four experimental groups were subjected to immunohistochemistry with one of the three anti-MHC antibodies (MHC-IIa, MHC-IIb and MHC-IId/x) for fiber typing, an anti-Zs-Green1 antibody for evaluation of the transfection success and with Alexa Fluor-405-conjugated BS-1 lectin for labeling of capillaries. The white circle in the merge tags an MHC-IIb/ZsGreen1 double-positive skeletal muscle fiber.

Baum et al.
